# Limited Top-Down Influence from Recognition to Same-Different Matching of Chinese Characters

**DOI:** 10.1371/journal.pone.0156517

**Published:** 2016-06-03

**Authors:** Jennifer Chang, Yifeng Zhou, Zili Liu

**Affiliations:** 1 Department of Psychology, University of California, Los Angeles, California, United States of America; 2 School of Life Sciences, University of Science and Technology of China, Hefei, Anhui, China; University of Akron, UNITED STATES

## Abstract

We investigated the extent to which recognition of Chinese characters influenced same-different matching performance that did not require recognition. In each experimental trial, two partially occluded characters were shown sequentially, and participants decided whether or not they were the same. The two characters were either both upright or both inverted and mirror-reflected. The participants’ Chinese reading fluency spanned the full range, from not knowing any characters to native speakers. The participants who could recognize some characters (defined as readers) were subsequently tested with character recognition in a naming task. Interestingly, although the readers’ recognition accuracies well correlated with their years of Chinese language schooling, they were uncorrelated with the matching accuracies in the same-different task with upright characters. The only indication of top-down influence was the readers’ higher accuracy in matching upright than inverted and reflected characters. However, the magnitude of this effect was small, to the extent that the average same-different accuracies were comparable for readers and non-readers alike. This small effect was further confirmed with native speakers in China, who should give rise to the largest possible effect. We conclude that top-down influence from character recognition was present but very limited, at least with the task and stimuli used.

## Introduction

One important question in human visual perception is how the visual system encodes an input stimulus into memory. This question is particularly important when the stimulus is impoverished, for example, when partially occluded or imbedded in noise, because imperfect stimuli are common-place. One might expect that the visual system, working from the stimulus input, first “perceptually completes” the shape rather than encoding the raw stimulus image as is. However, this completion cannot be completely certain. In this case, what a perceiver can do is infer the missing information from the regularities of objects in the world [1]. For example, using a partially occluded face image, Kersten (1987)[2] demonstrated that humans could use the luminance of the face region that surrounds a small occluder to infer the luminance of the face behind the occluder. As Kersten (1987) pointed out, humans could make reliable perceptual inferences in this case because objects in the world are shaped in such a way that their images carry redundant or correlated luminance information between neighboring regions. In other words, a missing pixel’s value in an image of a natural scene can be estimated, to a certain degree of accuracy, from the neighboring pixel values.

Another potential source that may aid this inference is familiarity with the object shapes. In the Kersten (1987) study, a face image was used that was partially occluded by small squares. Given that a human face was used, one may reasonably expect that inferring the value of an occluded pixel in a face image may also benefit from the perceiver’s familiarity with upright faces [3]. Indeed, such benefit was found in a visual memory study with partially occluded faces [4]. There, recognition sensitivity d’ was found to be better for upright than for inverted faces (see also [5]).

Faces, however, may not be the best class of objects to study the effects of familiarity. This is because, in addition to the controversy regarding whether or not faces are uniquely different from all other objects [6,7], all humans are experts of face recognition (except prosopagnosics). That is to say, there is practically no graded degree of expertise for faces. A continuous range of expertise is desirable because the degree of expertise and the behavioral performance on object perception can be then correlated. For this reason, text is commonly used as stimuli in the study of expertise. Pelli et al. (2009)[8] have further argued that using text is appropriate in the study of generic object recognition. The current study used Chinese characters to study the extent to which expertise with Chinese characters influenced perceptual performance of shape recognition.

The question whether recognition of text, faces, or other objects, facilitates behavioral performance in a perceptual task is important, because the finding will reveal the extent to which high-level object representation penetrates lower-level perceptual representation. Here, we use the terms of high- and low-level vision in the functional sense, but not in the anatomical sense of visual areas in the brain. Traditionally, object recognition is considered high-level vision that involves long-term memory representations of objects [9]. For example, naming is a typical task in object recognition research that by definition taps into semantic representation of an object [10]. In comparison, a same-different matching task need not require an object to be namable by the participant. For example, a participant who cannot read any Chinese characters can still determine whether or not two Chinese characters are the same. The research question here is: in a same-different matching task do participants who can recognize Chinese characters outperform those who cannot?

In the literature, readers and non-readers of Chinese characters have been shown to perceive differently in the following apparent motion study. Tse and Cavanagh (2000)[11] first presented a Chinese character that missed only one stroke, and therefore was recognizable by Chinese readers. The missing stroke was subsequently added all at once in an apparent motion sequence. Interestingly, this added stroke appeared to a Chinese reader to “shoot” from one end of the stroke to the other in the direction the stroke would have been written. In comparison, to a non-reader, this “shooting” motion direction was opposite and consistent with what low-level stimulus-driven cues would predict. This result was evidence that top-down influence from character recognition overrode bottom-up cues in motion perception. Familiarity with digits and letters was also found to strongly facilitate visual search [12]. This effect, however, was strongest when the background distractors were familiar (e.g., letter Z’s) while the target was unfamiliar (e.g., a mirror-reflected letter N).

Object recognition was also found to influence low-level distance discrimination between dots in three-space. Lu, Tjan, and Liu (2006)[13] used a number of dots that were presented in three-dimensions. They found that when participants recognized that two pairs of dots corresponded to the forearms of a point light human figure [14], discrimination between the two lengths became worse than before recognition, presumably because a human’s forearms are expected to be equally long. In a control experiment, after recognition, if the two distances were not expected to be equal, for example, the distances between the wrist and hip on the left and on the right side, the discriminability was unchanged from pre- to post-recognition.

Existing evidence suggests, therefore, that higher-level and long-term memory representations may indeed facilitate performance in a lower-level perceptual task, when the shapes used are recognizable. We tested this hypothesis in the current study.

Specifically, we used Chinese characters in a same-different matching task, and tested participants with a variety of Chinese character recognition ability. In addition, using a within-subject design, we also compared same-different performance between matching upright versus matching inverted and mirror-reflected characters. Surprisingly, we found little difference in performance between participants, and only a small difference between upright and inverted / mirror-reflected characters within those participants who could read Chinese. The readers’ same-different matching performance was also uncorrelated with their character recognition ability, even though their duration of Chinese language schooling was well correlated with their accuracy in a subsequent character recognition test. We conclude that the influence from the characters’ longer-term memory representation was very limited to their shorter-term representation in the same-different matching task.

## Experiment 1: Same-Different Matching and Recognition Tasks at UCLA

### Stimuli

Two hundred Chinese characters were used. Each character had no more than five strokes, whose traditional and simplified versions were also identical. That is to say, the same 200 characters are used everywhere in the world including mainland China, Hong Kong, Taiwan, and Singapore. This consistency in character shape and meaning eliminated the complication of comparing reading ability between participants from different parts of the world.

**Fig 1:**
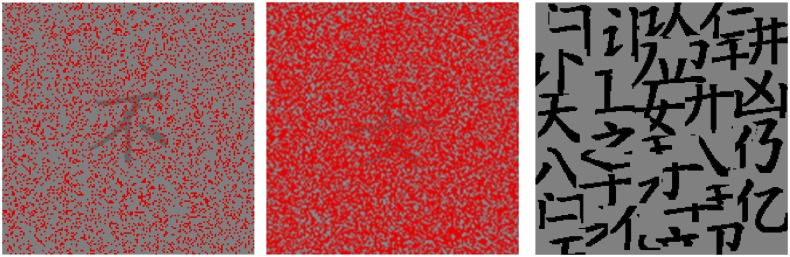
Left: an example stimulus image, with randomly distributed red occluding pixels occupying 20% of the image area. Middle: a different character that is occluded by red pixels occupying 60% of the image area. Right: a mask used in the same-different matching task.

Each character was dark gray (luminance: 28.7 cd/m^2^) and was approximately 16 × 16 mm^2^ in size. The character image was 46 × 46 mm^2^, with the background color being light gray (41.4 cd/m^2^). Hence, the Weber contrast of a character was 31%. Each character was partially occluded by red pixels that were randomly distributed over the entire image. The luminance of the red pixels was 12.6 cd/m^2^, with a color coordinate of (0.549, 0.329). Fig 1 shows an example character with 20% occluding red pixels, a second example character with 60% occluding red pixels, and a mask used in the experiment.

### Procedure

In each trial of the same-different matching task, the image sequence was as follows: the first character image (1000 ms), mask (200 ms), fixation (50 ms), the second character image (250 ms), mask (200 ms), and fixation. Participants decided whether the two characters were the same or different. No feedback was provided.

More specifically, each of the two character images was occluded by red pixels that occupied either 20%, 30%, 40%, 50%, or 60% of the image area, making a total of 5 × 5 = 25 possible occlusion combinations. The purpose of creating these 25 occlusion conditions was to span a wide range of difficulty levels, so that any possible top-down effects could be captured. Half of the 200 characters were randomly chosen to be in 100 “same” trials, with four characters in each of the 25 occlusion conditions. The 100 first images in these 100 “same” trials were then paired with the remaining 100 characters to make up 100 “different” trials, four pairs in each of the 25 occlusion conditions. In **⅕** of those “same” trials when the numbers of red pixels in the two character images were also the same, the distributions of the red pixels were identical so that the two stimulus images were exactly identical. The pairing, occlusion, and same or different assignment of the characters were otherwise randomized across participants. These 200 trials took about 15 min to complete.

The same 200 trials were then repeated in a randomized order, in a second block. This time, all characters were inverted and mirror reflected. For simplicity, we call this condition “inverted.” The order of these two blocks was counterbalanced between participants.

The experimental parameters were selected from a pilot study, such that participants who either could or could not read Chinese were still all able to perform the same-different task above chance yet under ceiling, ideally around 0.75 in proportion correct.

After the two blocks of the same-different matching task, the readers (defined as those participants who claimed to recognize some Chinese characters) were tested with their Chinese character recognition. An occlusion-free image of an upright character was shown on the computer monitor for 1000 ms, and the participant needed either to pronounce it correctly in Mandarin or to explain its meaning to the experimenter. Seventy-five characters were tested, which were randomly sampled from the available 200 characters. By sampling from the same 200 characters that were used in the same-different matching task, we aimed to make the measure of the recognition accuracy as relevant as possible to the same-different matching accuracy. The non-readers (defined as those participants who claimed not able to recognize any Chinese characters) were not tested with this recognition task.

### Participants

A total of 156 UCLA undergraduate students were recruited for course credits from the Psychology Department’s human subject pool. The only prerequisite for the recruitment was that participants had normal or corrected-to-normal visual acuity. Chinese reading ability was not mentioned in the recruitment. At the beginning of the experiment, participants were asked for their years studying Chinese. Among the 156 participants, 105 could read some Chinese, who are referred here as readers. Among these 105 readers, 37 grew up with Chinese as their native tongue and had learned to read and write Chinese; 47 were Chinese-Americans who had attended Chinese language schools in the US for various durations. The remaining 21 readers had learned Chinese characters as part of their own languages, for example, Korean or Japanese.

Fifty readers and 28 non-readers were randomly assigned to first run the block with upright characters. The remaining 55 readers and 23 non-readers ran in the opposite order.

### Apparatus

The experiment was conducted in a dark room with a calibrated Dell 17” computer monitor. The monitor resolution was 1024 × 768 pixels, and the refresh rate was 75 Hz. The computer’s CPU was Pentium 4 with a graphics card of RADEOM X300 (ATI Technologies Inc.). The participants viewed the stimuli binocularly, with a viewing distance of 57 cm. MatLab software (Math Works, Inc.) and Psychophysics Toolbox [15,16] were used.

### Ethics statement

The participants provided their verbal consent, due to the minimal risk nature of the experiments, to participate in all experiments reported in the current study. The participant consent was recorded together with the participant’s initials. These study protocols were approved by the Office of Human Research Protection Program, University of California Los Angeles; and by the ethics committee, University of Science and Technology of China. The protocols were in agreement with the Declaration of Helsinki.

### Results

Since there were 105 participants who could read some Chinese, and 51 participants who could not read any Chinese, we divided the 105 readers into two groups: more and less experienced readers. The division was based on the accuracy in the recognition test of the 105 readers after the same-different task. Two criteria were used: (1) the division should be as even as possible, and (2) participants with tied scores should be grouped together. As a result, 54 participants were grouped as more experienced (recognition accuracy in proportion correct ≥ 0.52) and 51 as less experienced readers (recognition accuracy ≤ 0.51).

**Fig 2:**
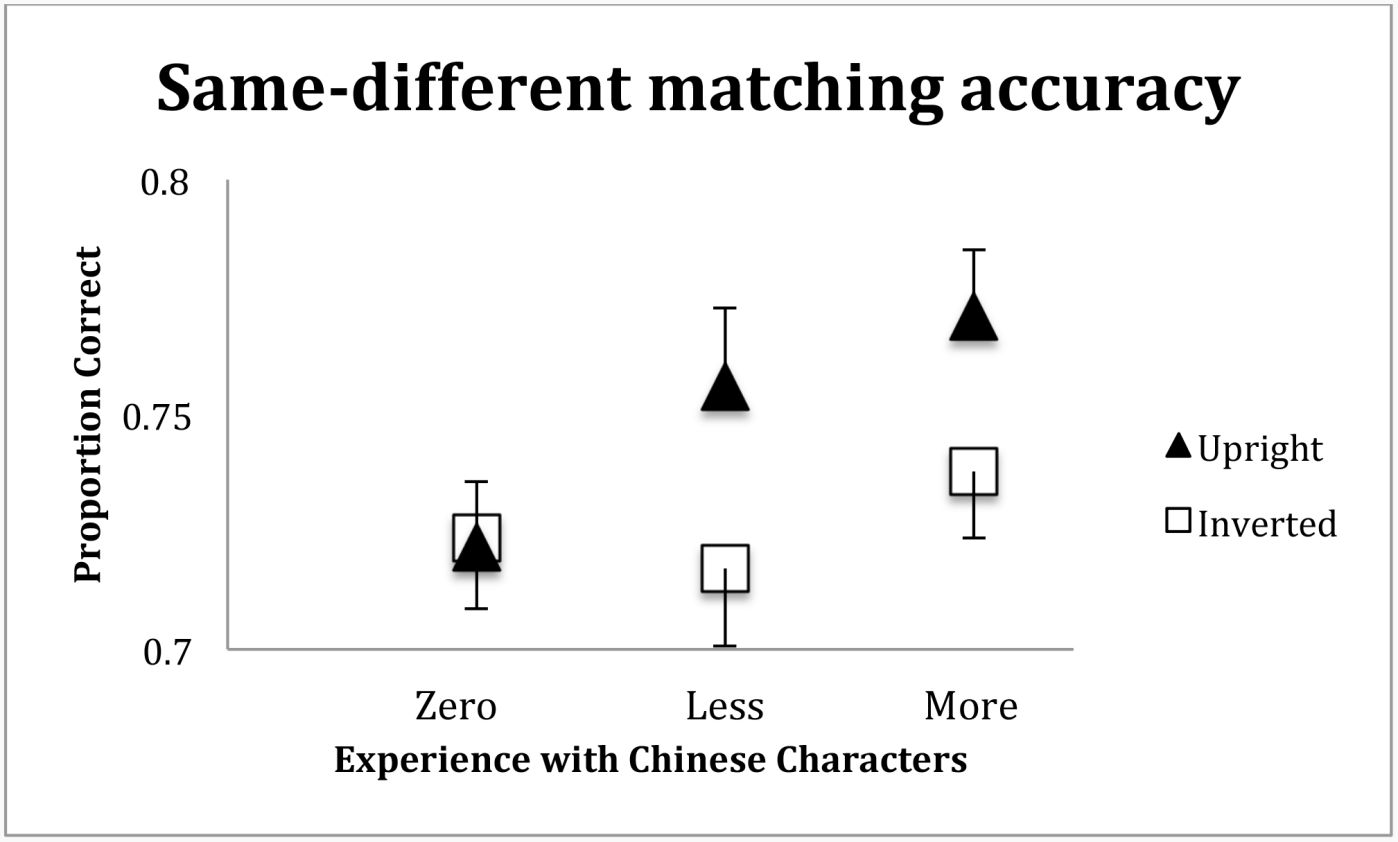
Proportion correct in the same-different matching for the zero, less, and more experienced participants, and for inverted and upright characters. The error bars represent standard error of the mean.

Since our primary interest was the influence of character recognition on lower-level same-different matching, we collapsed the 25 occlusion conditions and calculated the accuracies in proportion correct in the same-different task. Fig 2 shows the data. ANOVA was used to analyze the accuracy data, with character orientation (upright, inverted) and experience (zero, less, and more) as the two main factors. The main effect of character orientation was significant (accuracies = 0.74 and 0.73): F(1, 153) = 24.39, p << 0.001. The main effect of experience was not significant (0.72, 0.74, 0.75): F(2, 153) = 1.21, p = 0.30. The interaction effect was significant: F(2, 153) = 7.03, p = 0.001. This interaction effect means that, for the 105 readers, their same-different matching accuracy was higher for the upright than for the invert characters (0.76, 0.73). The interpretation of this interaction effect is supported by the following more detailed analysis.

As expected, the same-different matching accuracies for the 51 non-readers were the same (0.72, 0.72) for upright and inverted characters alike. With the inverted characters, a detailed analysis revealed that the 105 readers shared comparable same-different matching accuracies with the 51 non-readers (0.73, 0.72; t < 1). This means that the readers showed little top-down advantage in matching inverted characters than the non-readers. Consequently, the difference between matching upright and matching inverted characters could serve as a measure of the top-down effect. With the upright characters, the 105 readers were slightly more accurate than the 51 non-readers (0.76, 0.72), with the t-test just reaching statistical significance after Bonferroni correction (t = 2.36, p = 0.02). This means that matching upright characters was slightly more accurate for the readers than for the non-readers.

Taken together, the results above suggest that, on one hand, upright character recognition indeed facilitated the lower-level perceptual task of same-different matching. On the other hand, however, this facilitation from character recognition was surprisingly small in magnitude. Perhaps the most important evidence for this small facilitation is that the main effect of experience was not significant, despite the reasonably large number of participants. Numerically, the average accuracies in the same-different task for the 105 readers and 51 non-readers differed only by 0.03 (0.75 vs. 0.72). This difference was not statistically significant.

**Fig 3:**
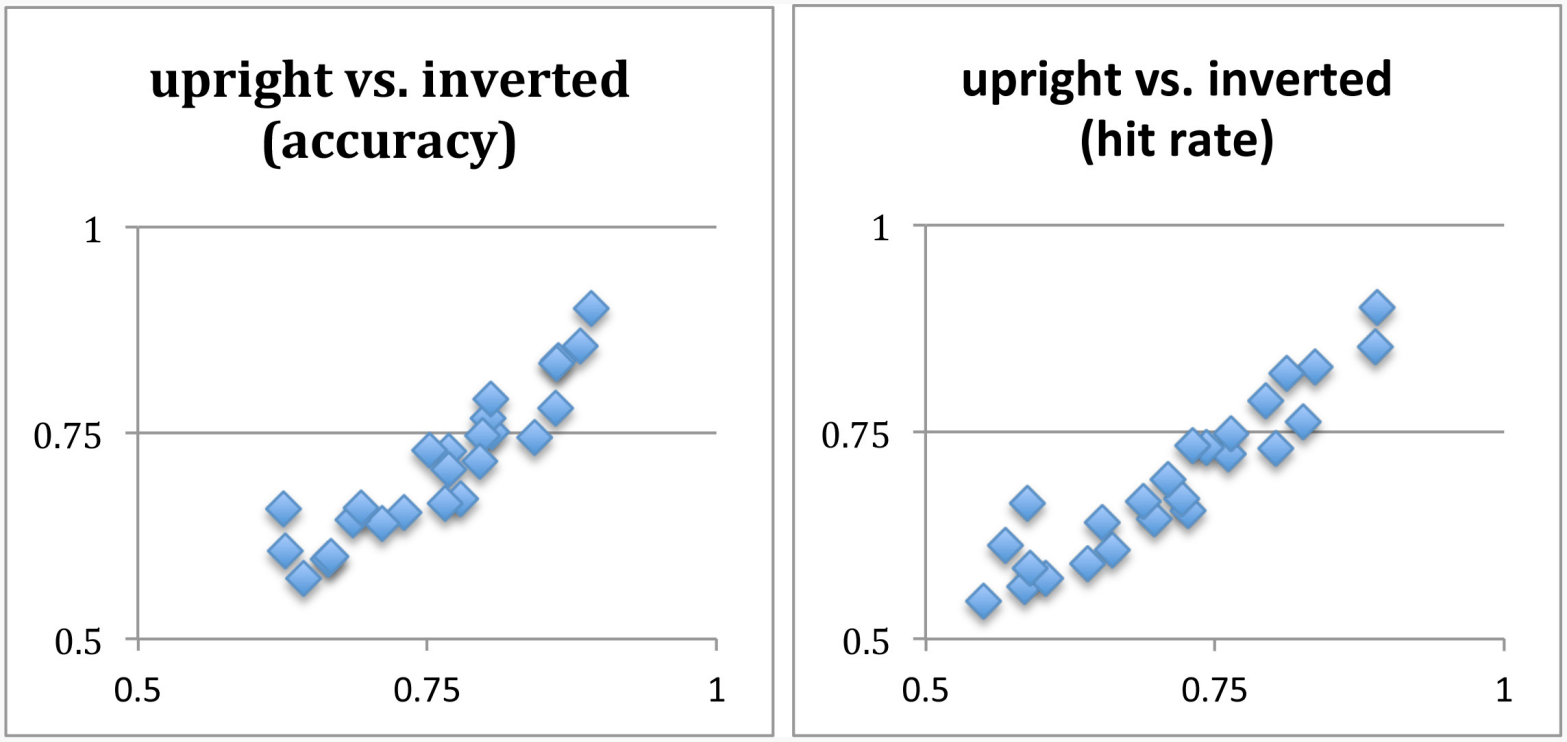
Scatter plots of upright (x-axis) vs. inverted (y-axis) same-different accuracies in proportion correct (left panel) and hit rates (right panel). Each panel shows the 25 occlusion conditions, each of which was averaged from the 105 readers.

We wondered whether or not this weak influence was due to the following possibilities. At the minimal occlusion, the characters were easily visible such that the top-down influence was unnecessary. At the maximal occlusion, when the characters were barely visible, it could be that recognition was difficult so that participants had to rely on low-level features to perform the same-different matching. As a result, the top-down facilitation may be more effective in the medium range of occlusion conditions. In order to test these possibilities, we plotted the proportion accuracies of the upright and inverted characters for the 25 occlusion conditions in a scatterplot (Fig 3, left), averaged from all 105 readers. There was little evidence that medium ranged occlusion conditions gave rise to greater upright-inverted difference than the two extreme occlusion conditions. The linear correlation coefficient was 0.92, and the linear slope was 0.96. These numbers suggest that the medium range occlusions did not give rise to substantially greater upright-inverted accuracy difference than the extreme occlusions.

We also wondered whether or not the top-down influence would be more pronounced in the ‘same’ trials when the two characters in a trial were identical. This was a possibility because, in a ‘different’ trial, participants might resort to low-level image features to ascertain that the two characters were different, without relying on recognition of the characters. In order to verify this, we made a similar scatterplot, except this time the hit rates (defined as responding “same” when the two characters were the same) across the 25 occlusion conditions were used, rather than the overall proportion corrects. As shown in Fig 3-right, there was little evidence that medium ranged occlusions gave rise to greater top-down influence either. The linear correlation coefficient was 0.94, and the linear slope was 0.91. In summary, since the proportion corrects and hit rates covered the entire range from 0.5 to 1, they covered the full range of task difficulty too. In this entire range, there was little evidence that the top-down influence was pronounced in any difficulty level.

**Fig 4:**
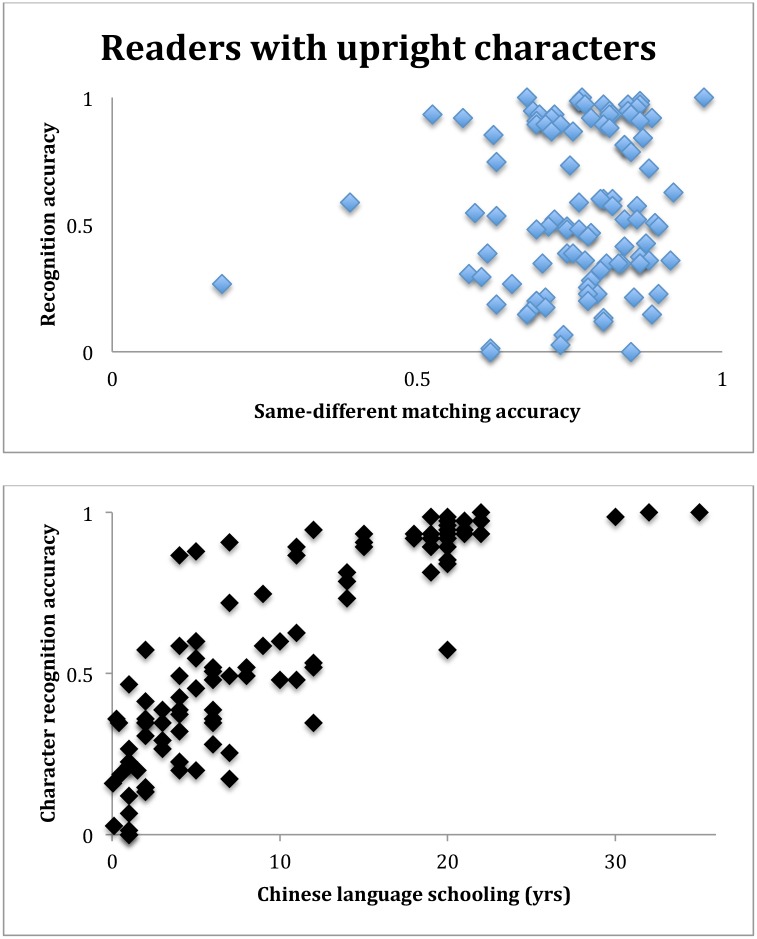
**Top**: scatter plot for the 105 readers of the same-different matching accuracies with upright characters and the recognition accuracies in the subsequent character recognition task. There was little correlation between the two measures. **Bottom**: scatter plot of the 105 readers’ years of learning Chinese and accuracies in the character recognition task. The correlation (r = 0.85) was statistically significant.

The weak influence can be further illustrated in the following correlational analysis. For the 105 readers, their accuracies in proportion correct in the same-different matching of upright characters were correlated with their subsequent character recognition accuracies. This correlation was not significant: r = 0.13, t(103) = 1.36, p = 0.18. In order to put this correlation in perspective, a similar correlation was performed, except this time the characters in the same-different matching task were inverted. This correlation was not significant, as expected; but the correlation coefficient was comparable to that for the upright characters: r = 0.12, t(103) = 1.23, p = 0.22. These small and insignificant correlations could not be due to the possibility that the recognition accuracy was a poor measure of character recognition ability, because the character recognition accuracy correlated well with the 105 readers’ years of schooling in learning Chinese: r = 0.85, t(103) = 16.17, p << 0.001 (Fig 4).

**Fig 5:**
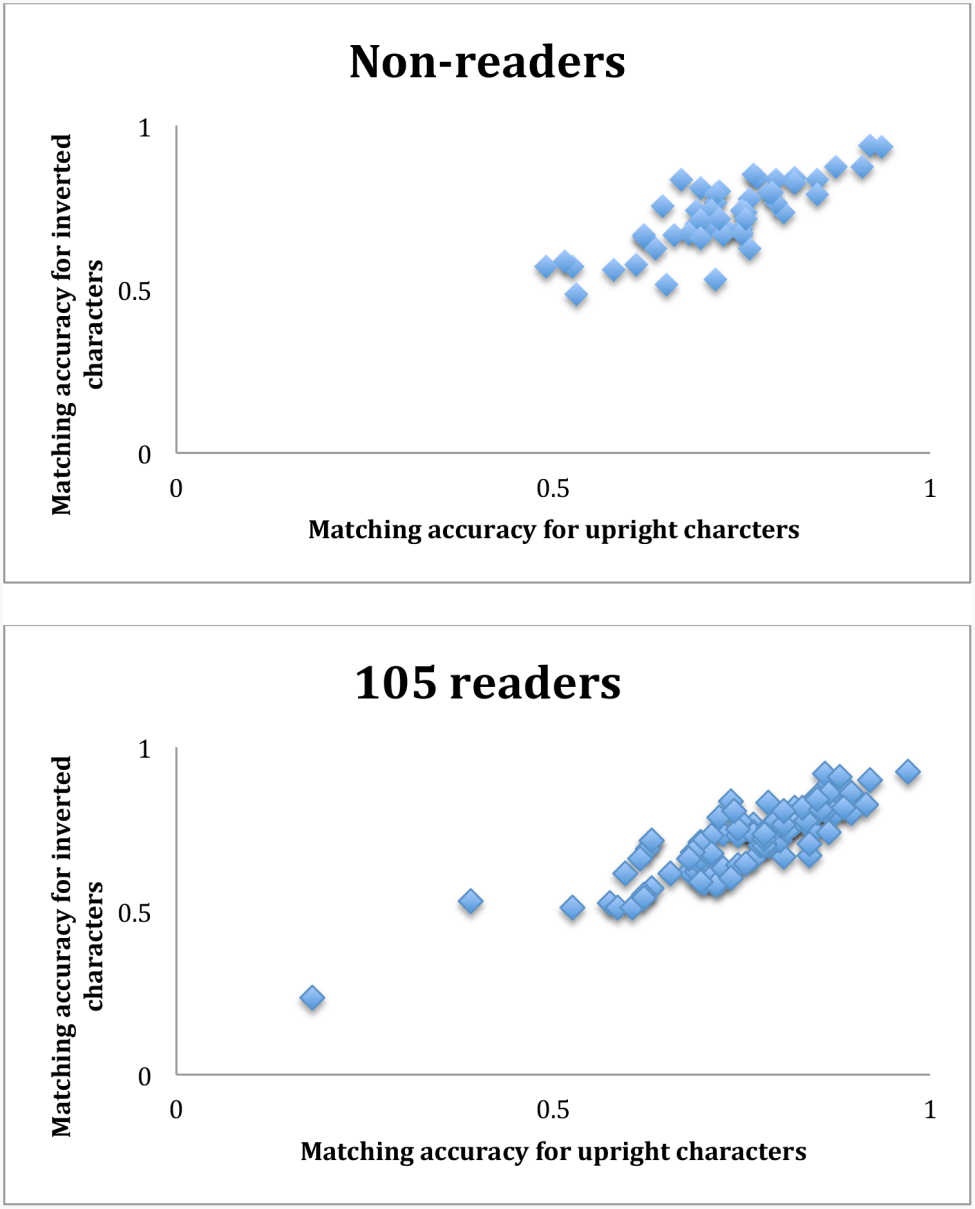
Scatter plots of proportion correct accuracies in the same-different matching task for upright and inverted characters for the 51 non-readers (top), and for the 105 readers (bottoms). The two correlations were statistically significant and comparable to each other.

The above correlation analysis can also be appreciated if we look at the correlation of same-different matching accuracies between the upright and inverted characters for the 51 non-readers: r = 0.81, t(49) = 13.82, p << 0.001. This high correlation certainly had nothing to do with character recognition because these participants could not read any Chinese. In comparison, when the same correlation was performed for the 105 readers, the correlation coefficient was r = 0.87, t(103) = 18.16, p << 0.001 (Fig 5). This means that a participant’s same-different accuracy matching upright characters could be well predicted by the same participant’s accuracy matching inverted characters, when the inverted (and reflected) characters were very difficult, if not impossible, to recognize [17]. These correlations indicate that the participants’ performance in the same-different matching task was largely determined by their ability to match occluded characters, no matter if the characters were upright or inverted, and associated with character recognition only to a small degree.

**Fig 6:**
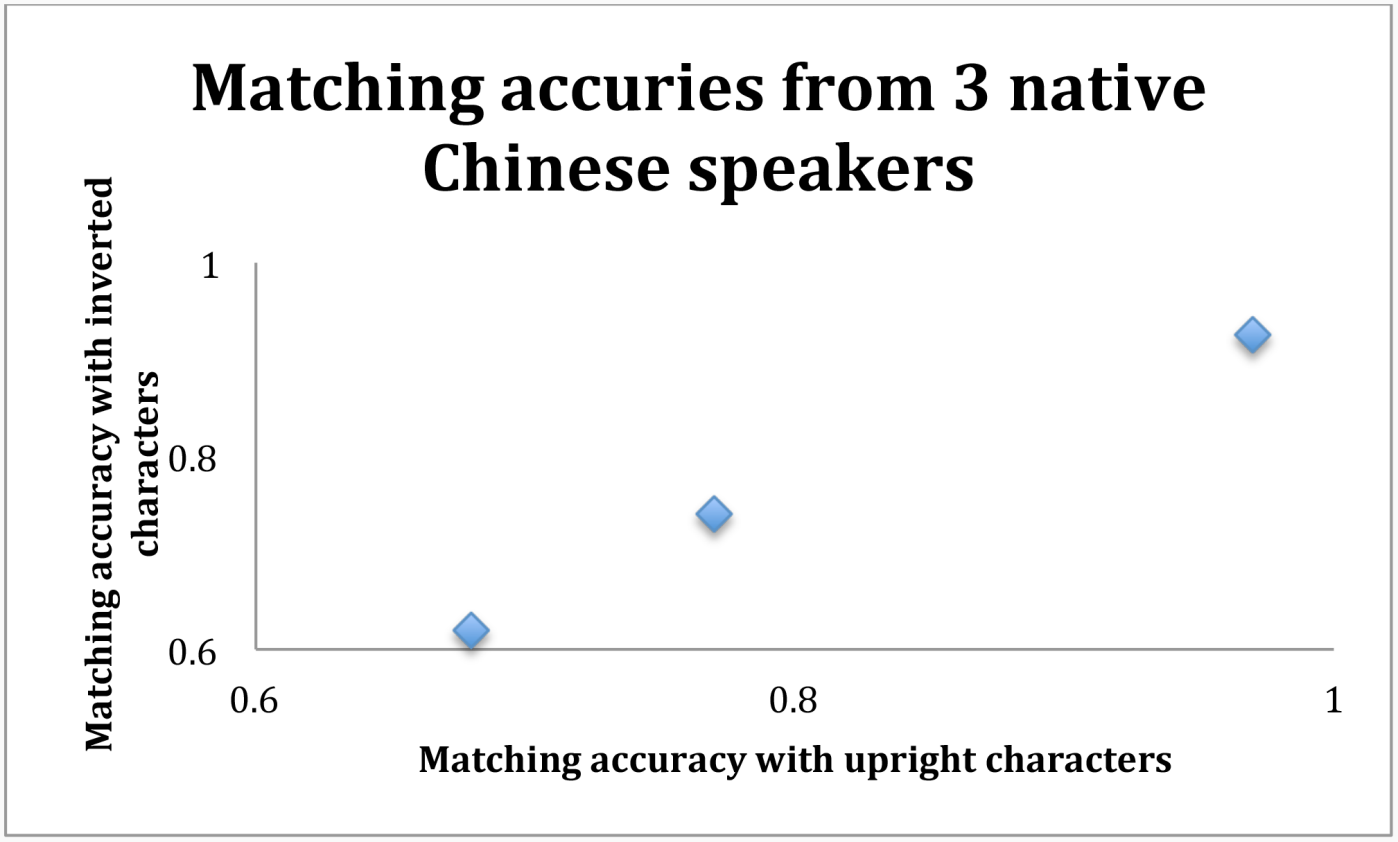
Same-different matching accuracies with upright and inverted characters, from three native Chinese speakers who scored perfectly in the character recognition test. These three native speakers’ same-different matching accuracies were nevertheless not perfect.

While analyzing the correlations, we also noticed that three (and only three) readers scored perfectly in their character recognition test. These three readers turned out to be native speakers who grew up in China. To our surprise, however, their same-different matching accuracies were not perfect even with the upright characters (Fig 6). Nevertheless, since these were only three participants, no firm conclusions with meaningful statistics could be made. We therefore decided to test native speakers in China under identical conditions to confirm this result from the three native speakers at UCLA.

There was also the following reason for us to test native speakers in China. There was a potential confound on subject selection at UCLA, because the more and less experienced readers were not randomly sampled. Among those participants who learned Chinese as a second language, some may be better at memorizing Chinese characters than others. Therefore learning Chinese could be easier for them. As a result, these participants might continue to learn Chinese longer and became our more experienced readers. In other words, better recognition of Chinese characters might be correlated with better memorization of Chinese characters, among the UCLA participants. After this confounding factor is taken into consideration, the slightly higher accuracy (0.75 vs. 0.74) in the same-different matching for the more experienced than the less experienced readers might be further reduced. In order to remove this confound, we tested in the next experiment native speakers who, like those at UCLA, were also college students.

## Experiment 2: Same-Different Matching with Native Speakers in China

The purpose of this experiment was to determine whether or not, under identical experimental conditions, native Chinese speakers gave rise to comparable same-different matching accuracies as the 105 readers in Experiment 1. An even more important purpose was that, by testing native speakers, any influence of character recognition on the lower-level same-different matching task would be maximized. This prediction was based on the results from Experiment 1 (Fig 2) that same-different matching accuracies were comparable for all participants with inverted characters (readers: 0.73, non-readers: 0.72), whereas the readers showed a higher matching accuracy than the non-readers with upright characters (readers: 0.76, non-readers: 0.72), which was confirmed by the interaction effect in the ANOVA.

Twenty students, 10 men and 10 women, were recruited from the Chinese University of Science and Technology, Hefei, China, with adherence to the Declaration of Helsinki. Five male and five female participants first ran 200 trials with upright characters. This was followed by the same 200 trials in a different order, and the characters were all inverted and mirror-reflected. The remaining 10 participants ran the experiment in the opposite order. Recognition of the characters was not formally tested, because our informal test indicated clearly that all characters were recognizable by these college students.

All the experimental parameters, including the luminance values, were the same as in Experiment 1. The monitor was a 17” Sony G220, and the computer used a graphic card Nvidia GTX460.

### Results

**Fig 7:**
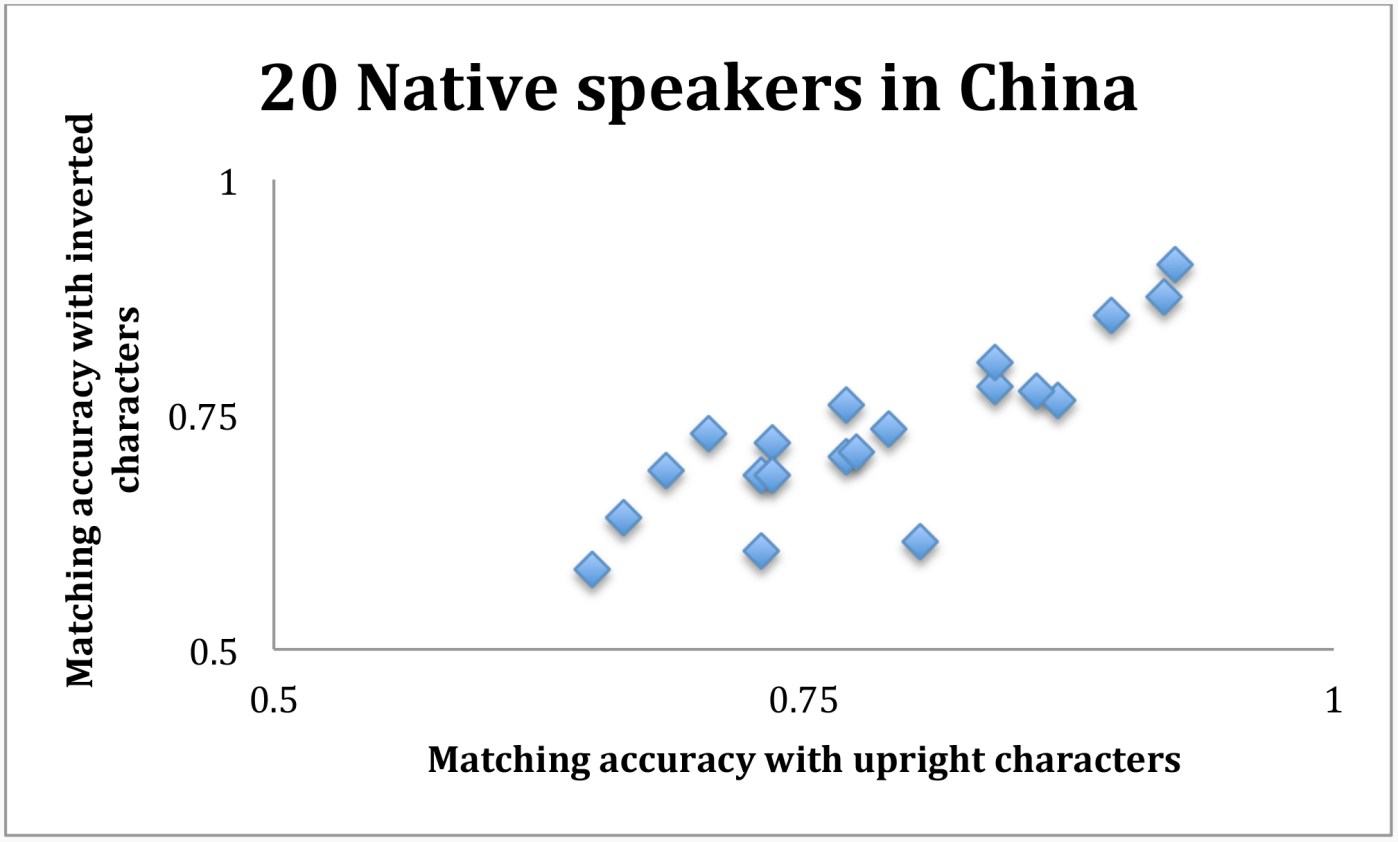
Scatter plot from the 20 native speakers of their same-different matching accuracies with upright and inverted characters. The correlation coefficient was 0.84.

It turned out that the average same-different matching accuracy was 0.78 for the upright characters, and was 0.73 for inverted characters (note that in Exp.1, the accuracy for the inverted characters was 0.72 for non-readers and 0.73 for readers). This difference was statistically significant: t(19) = 4.96, p << 0.001. However, this difference was small in magnitude, and comparable to the (0.77, 0.74) accuracies from the 54 “more experienced” readers in Experiment 1. The small difference (0.78, 0.73) could not be due to any ceiling effect, because the average performance was only 0.76. The correlation coefficient was 0.84 between matching upright and matching inverted characters for these 20 participants (Fig 7). This correlation was consistent with Experiment 1, again suggesting that a native speaker’s same-different matching performance with upright characters could be well predicted by the same person’s performance with inverted characters.

The evidence thus far implicated only a small role of higher-level recognition of Chinese characters in the lower-level perceptual task of same-different matching. However, this effect was obtained under only one set of experimental parameters. The extent to which this effect would persist under different conditions remained unknown. The next experiment tested whether the effect would be larger if the stimulus contrast was doubled. Since this top-down influence was expected to be stronger for native speakers, we again tested native speakers.

## Experiment 3: Testing Native Speakers with Higher Stimulus Contrast

In the two experiments above, the stimulus contrast (before red pixels were added) was 31%. In this experiment, we doubled the contrast to make the same-different matching task easier, in order to check whether or not the performance difference between upright and inverted characters would be bigger. The same setup was used as in Experiment 2, except the computer graphics card was an Nvidia 9800GT. The luminance of the Chinese characters was 1.68 cd/m^2^, and its background was 4.63 cd/m^2^, giving rise to a contrast of 64%. The luminance of the red pixels was 2.08 cd/m^2^.

Twenty-one fresh students, 11 men and 10 women, were recruited in the same way as in Experiment 2. Eleven participants (including five women) were tested in the same-different task with the upright characters first, and the remaining 10 participants were tested with the opposite order.

**Fig 8:**
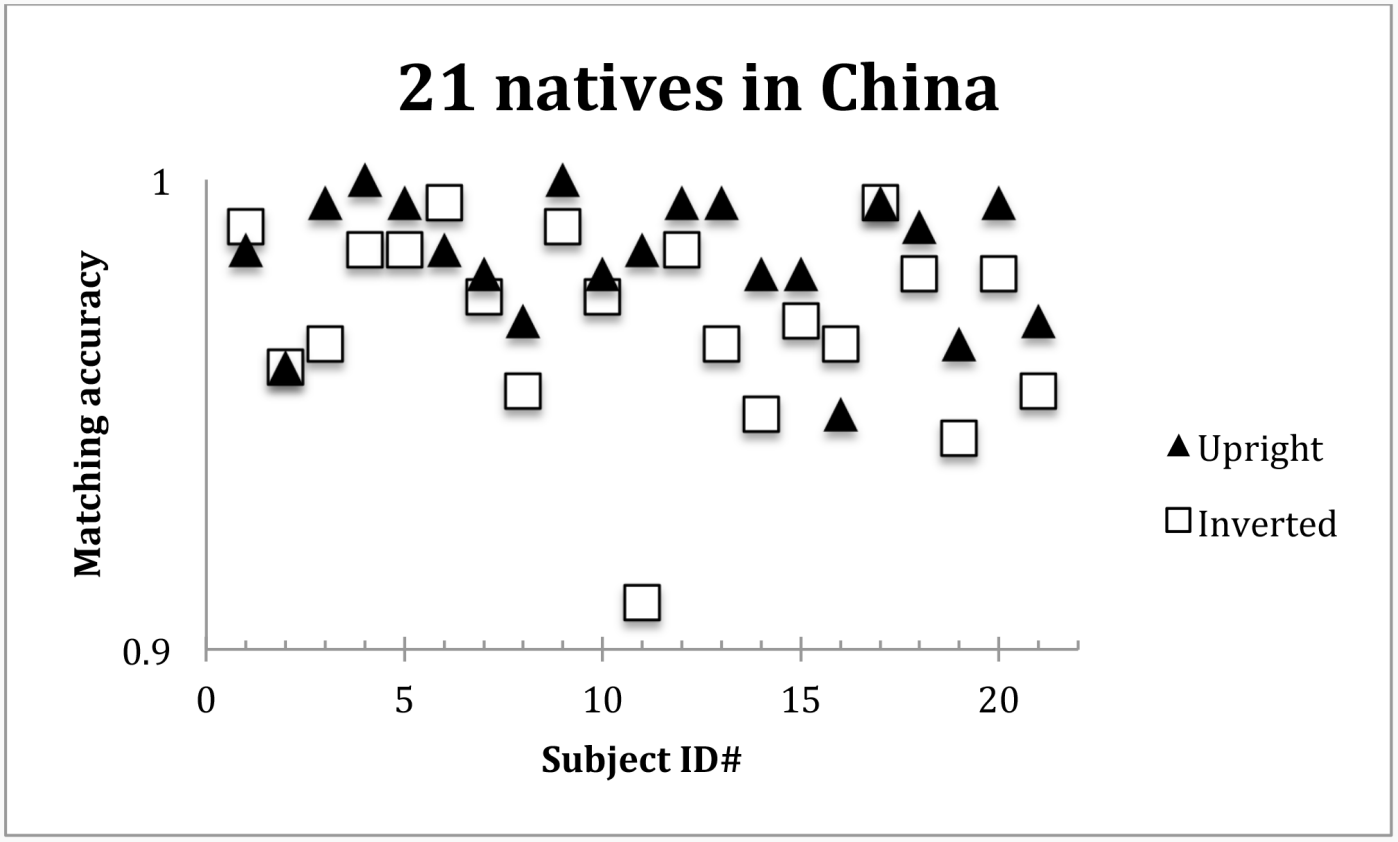
Proportion correct accuracies for the 21 native Chinese speakers, for the upright and inverted characters in the same-different matching task. All 21 participants except No.1 and No.16 showed a higher accuracy for the upright than for the inverted characters. The average difference was statistically significant, but was only 0.01 in proportion correct.

It turned out that the same-different matching accuracies in proportion correct were nearly perfect: 0.98 for upright characters, and 0.97 for inverted characters (Fig 8). The difference between these two numbers was statistically significant, t(20) = 3.23, p < 0.005. Obviously, however, the magnitude of the effect size was not bigger than in the previous experiments.

## Experiment 4: Comparing with 120 Non-Readers at UCLA

Thus far, 51 non-readers had been tested. Namely, the number of participants who could not read any Chinese was 51. Although 51 participants was already a reasonable number in a visual psychophysical study, it was still much smaller than the 146 readers tested. In this experiment, we aimed to compare data from readers and non-readers with data from more non-readers.

In the Experiment 4 of Lu and Liu (2009)[18], 120 non-readers and 29 readers were tested in a same-different matching task with upright characters at UCLA. The accuracy result was never reported for the 120 non-readers (but the hit rate was reported), and no result was reported for the 29 readers. Here we report the same-different matching accuracies with 200 trials per participant. In this experiment, the luminance of the Chinese characters was 41 cd/m^2^, and the luminance of the background was 56.1 cd/m^2^. The resulting Weber contrast was 27%, as compared with the 31% in Experiments 1 and 2, and 64% in Experiment 3. This experiment was otherwise identical to the first three experiments above.

**Fig 9:**
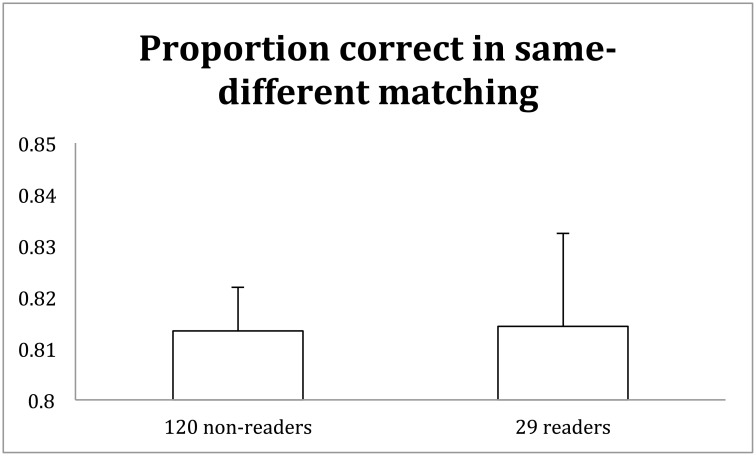
Proportion correct in the same-different task with upright Chinese characters by 120 non-readers and 29 readers of Chinese. The accuracies of the two groups were identical. Error bars are standard errors of the mean.

It turned out that the same-different matching accuracy was the same for the 29 readers and 120 non-readers alike (0.81, 0.81) (Fig 9). In Experiment 1, the 105 readers were slightly more accurate in matching up upright characters than the 51 non-readers (0.76, 0.72). This small discrepancy between the two experiments reflected, on one hand, the sampling variation of participants and the varying sample size. For example, the 29 readers were not tested in a separate character recognition test, and their number of years of Chinese schooling was not assessed. On the other hand and broadly speaking, the data from these two experiments consistently indicated that any facilitation from recognition to same-different matching of Chinese characters was very limited.

## Discussion

Characterizing the nature of internal shape representations is one of the most important problems in visual perception. The problem is also difficult when the stimulus shape is, for example, embedded in a clustered background, rotated in depth or in the image plane, in low contrast, or partially occluded.

In this study we have considered two possible ways in which a shape’s representation may be internally reconstructed. The first is based on the fact that objects are not shaped arbitrarily but obey certain regularities. For example, even for human made Chinese characters, each stroke follows the simple rule of good continuation, probably because the strokes originally need to be produced by a paintbrush in a smooth motion. The second and independent way of inferring an underlying shape representation is based on familiarity. For example, in the example of Tse and Cavanagh (2000)[11], a participant knowing how to write Chinese perceived the illusory motion in the opposite direction as compared to a participant not knowing any Chinese.

In the current study, the first character image was shown for 1000 ms, which was long enough to involve long-term memory. The presentation time of the second character image, 250 ms, was also long enough for long-term memory to exert its influence. Our main result, however, is that although the top-down influence was evident, the effect size of this influence was small. Given the reasonably large number of participants, this overall effect and its magnitude were robust.

Our evidence of top-down effect was that the readers were more accurate matching upright than matching inverted characters, whereas non-readers showed no difference, and readers and non-readers little differed in matching inverted characters. But this effect was very limited in the sense that the average accuracy of the same-different matching was similar for readers and non-readers alike. In other words, the ability to recognize the characters could not give rise to a higher same-different matching accuracy.

This small effect size could not be due to any ceiling effect, because in Experiment 1 the average performance was 0.75. The small effect could not be due to a subject selection process that resulted in participants knowing too much or too little Chinese. This is because the participants’ ability recognizing Chinese characters covered the entire range of recognition ability. It is also important to note that there was no correlation between the reading ability and the effect size.

In Experiment 1, the 54 more experienced readers’ same-different matching accuracies were 0.77 for the upright, and 0.74 for the inverted characters. The relative difference was 4% ((0.77–0.74) / 75.5). If we consider all 105 readers, the accuracies were 0.76 and 0.73, giving rise also to a relative difference of 4%. In Experiment 2, the 20 native speakers’ same-different matching accuracies were 0.78 and 0.73, giving rise to a relative difference of 7%. How is this effect size compared to the results in the literature as reviewed in the Introduction?

In [11], seven out of 10 Chinese subjects (70%) saw the apparent motion in the opposite direction as non-Chinese subjects. In Wang, Cavanagh, and Green (1994)[12], when the 2’s and 5’s were rotated by 90°, the search speed was 82 ms / item. When the target was a mirror reflected N or Z, and the distractors were regular Z’s or N’s, the search speed became 1.5 ms / item. The relative change was 193%. In [13], distance discrimination sensitivity between two dot pairs was measured by a psychometric function, which was fitted as a cumulative Gaussian function. The standard deviation of this Gaussian function increased from 3.36 to 4.28 mm, after recognition of the point light human figure. The relative change was 24%. Finally, the study closest to the current study was [18], who used a same-different matching task with upright and inverted faces. As in the current study, the face images were also partially occluded by red pixels. There, the relative effect size was 19% (mean proportion correct: 0.76). In the current study, the relative effect size was 7% (the mean proportion correct was also 0.76).

Although the estimates of relative effect size from different experiments were mostly non-comparable with each other, the comparison between the current study with [18]’s face experiment is meaningful because the same experimental methods and metrics were used in the measurements. The effect size of the current study was less than half of that in the face experiment in [18].

One might be concerned with the data analysis method, namely ANOVA, which was used in analyzing proportion corrects from the categorical same-difference responses [19]. Technically, the concern is that while a proportion correct measure is always between 0 and 1, a confidence interval may however cover a range smaller than 0 or greater than 1. We believe, nevertheless, that this concern is unlikely to be critical for our conclusions. The most important reason is that the top-down effect size was completely independent of any analysis method. In addition, even if we consider the smallest effect size in the current study (0.98 vs. 0.97, Exp.3) with one of the smallest sample size (N = 21), the effect remained statistically significant by using the noncontroversial binomial test (19 out of 21).

Was the weak top-down influence due to our selection of Chinese characters that all had five strokes or less? We believe that this was unlikely for the following reasons. We selected these Chinese characters in a pilot study in order for participants who could not read any Chinese to be able to do the same-different matching task and without feeling overwhelmed. However, one possible concern was that the characters may be too simple and the task too easy, so that any top-down influence became unnecessary. We addressed this possibility by using a range of red occluding pixels that made the same-different matching task more difficult. Indeed, the resultant proportion correct of the task performance covered the full range from chance to ceiling, but the magnitude of the top-down influence was comparable and small across this entire range. This nearly uniform effect size argues against the possibility that the task was too easy for the top-down influence to be effective. We acknowledge, nevertheless, whether or not stronger top-down influence can be found using more complex Chinese characters remains an empirical question for future research.

Another possible argument is that, with the Chinese characters used, it is perhaps easy to tell when two characters were different. Hence, the top-down influence was unneeded in all the ‘different’ trials. We addressed this possibility by looking at only the ‘same’ trials. Here, the two images in each ‘same’ trial had different occlusions 80% of the time. One needs to carefully compare the two images in order to determine whether or not the two embedded characters were the same. Hence, one might expect to be able to more likely find the presumably more pronounced top-down influence. However, when we considered only the hit rates, the magnitude of the hit rate difference between upright and inverted characters was not greater than when the overall proportion correct was considered. We conclude therefore that the choice of the characters was unlikely to be responsible for the limited top-down effect.

If the top-down influence on the representation of the partially occluded characters was small, what might underlie the participants’ performance with these impoverished word stimuli? A prior study on identifying familiar English words showed that participants could not use a word as a whole pattern to recognize it [20]. Instead, simpler features at the letter or sub-letter levels were detected independently, thus bottlenecking the recognition process. This analytic, as opposed to holistic, account has nevertheless been challenged recently. For example, in visual word recognition, Houpt, Townsend, and Donkin (2014)[21] used response time based measure of efficiency to demonstrate that word recognition was more efficient than independent feature processing would predict. In visual object recognition, Bar (2003)[22] showed that a low spatial frequency version of the stimulus input (or a blurred image) could be projected rapidly from early visual areas in the brain to the prefrontal cortex. This low special frequency “image” is, by definition, a representation about the object and its context as a whole, rather than as parts. Bar (2003) further hypothesized that the representation triggered at the prefrontal cortex served as top-down “initial guess” of what the object might be and interacted with the bottom-up signals for recognition.

Our study was not designed to test whether Chinese characters were recognized by independent processing of features or parts. We speculate that it is possible that participants, readers and non-readers alike, primarily relied on the Gestalt law of good continuation to detect each stroke. Yet it is possible that readers were more efficient than non-readers as well as more efficient with upright than inverted characters. It is hence conceivable that the readers’ coding of the characters in the same-different matching was more global or holistic than independent feature processing, since they could recognize the characters. On the other hand, however, this extra aspect of the coding must be very limited given how small a role the top-down influence played.
